# Supplementary material for: Structure-Function Analyses of New SARS-CoV-2 Variants B.1.1.7, B.1.351 and B.1.1.28.1: Clinical, Diagnostic, Therapeutic and Public Health Implications
Source: Viruses. 2021 Mar 9;13(3):439. doi: 10.3390/v13030439 (PMC8000172; doi:10.3390/v13030439)
Supplement: Supplementary file 1 [file viruses-13-00439-s001.pdf]

Supplementary Information

Structure-function analyses of new SARS-CoV-2 variants B.1.1.7 and B.1.351: Clinical, diagnostic, therapeutic and public health implications

Jasdeep Singh [1], Jasmine Samal [2], Vipul Kumar [3], Jyoti Sharma [2], Usha Agrawal [2], Nasreen Z. Ehtesham [2], Durai Sundar [3,*], Syed Asad Rahman [4,*], Subhash Hira [5,*] and Seyed E. Hasnain [3,6,*]

Supplementary Figures

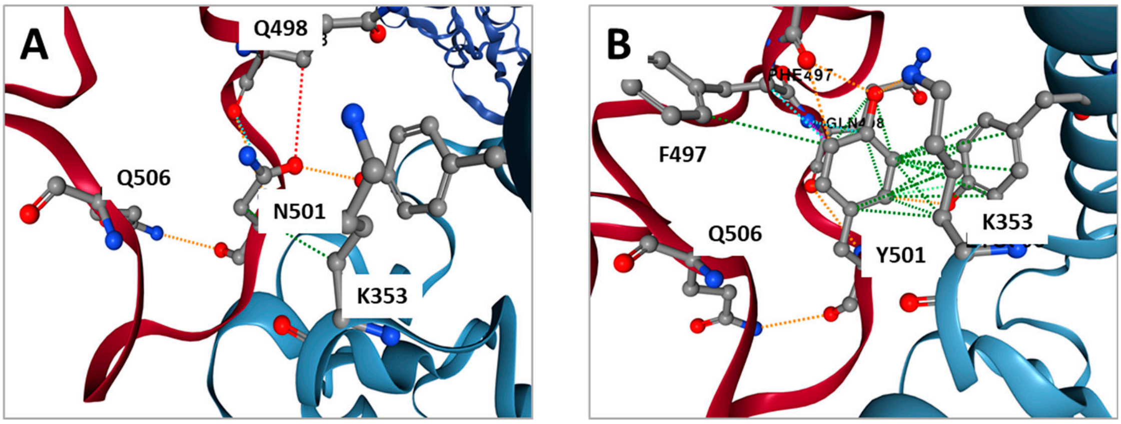

Figure S1. Structural localization of mutations in S protein (A) Residue interaction network at RBD (Brown)-ACE2 (Blue) interface for wild type N501 variant. (B) Residue interaction network at RBD (Brown)-ACE2 (Blue) interface for mutant Y501 variant. Color codes: H-bonds (red), Polar H-bonds (orange), VdW (light blue), Aromatic (light green) and Ring-ring interactions (brown).

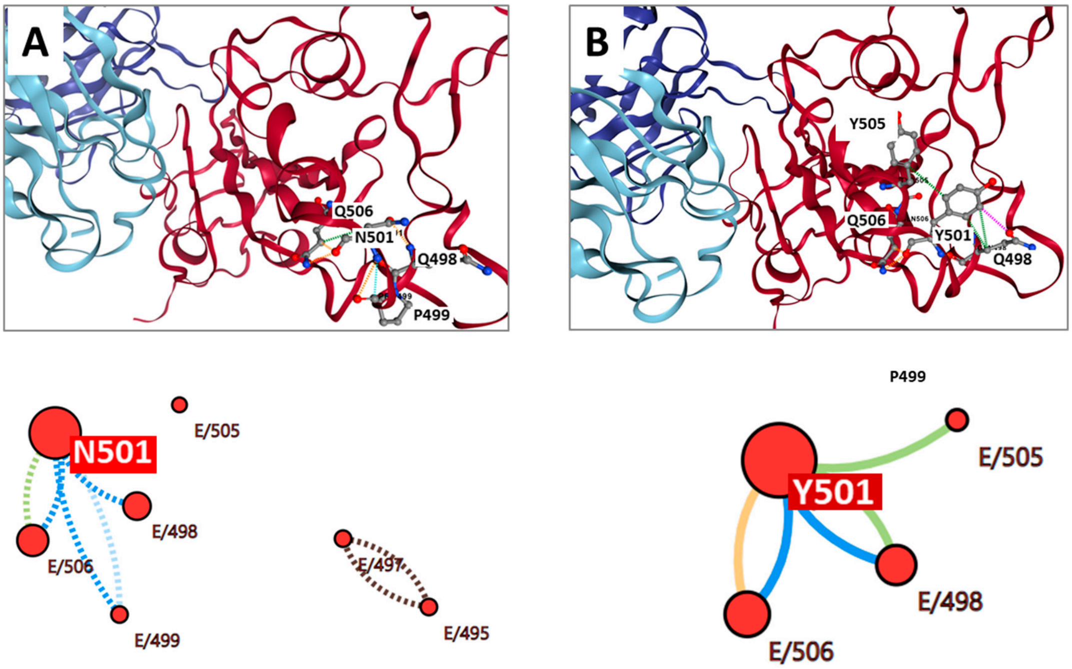

Figure S2. (A) Residue interaction network at RBD (Brown)-CR3022 (SARS-CoV-2 neutralizing monoclonal antibody, Blue) interface for wild type N501 variant. Panel below shows detailed interaction of N501 with other residues of RBD of S protein (marked as Chain E, Dashed lines). (B) Residue interaction network at RBD (Brown)- CR3022 (Blue) interface for mutant Y501 variant. Panel below shows detailed interaction of Y501 with other residues of RBD of S protein (marked as Chain A, straight lines). Color codes: H-bonds (red), Polar H-bonds (orange), VdW (light blue), Aromatic (light green) and Ring-ring interactions (brown). N501 or Y501 is not involved in direct interaction with CR3022 antibody.

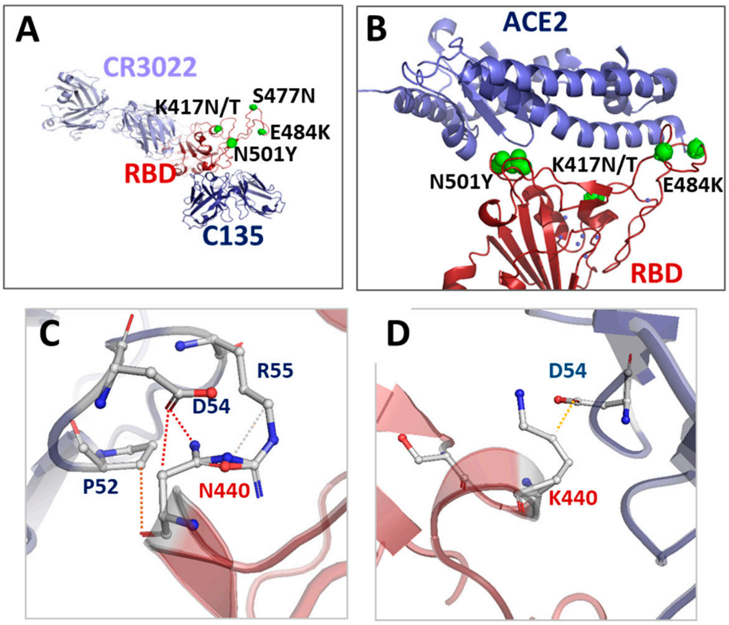

Figure S3. (A) Interaction interface between RBD (Brown) of S protein of SARS-CoV-2 and ACE2 (Blue). (B) Distinct interaction interface (epitope) between RBD (Brown) and C135 (Dark Blue) and CR0322 (Light Blue) antibodies. K417N/T, S477N and E484K mutations occur outside both RBD-antibody interfaces (shown as green spheres). (C) Residue interaction network of N440 (RBD, Brown) with residues of C135 antibody (Blue). (D) Residue interaction network of K440 (RBD, Brown) with residues of C135 antibody (Blue). Strong H-bonds are shown as red and weak H-bonds as orange.

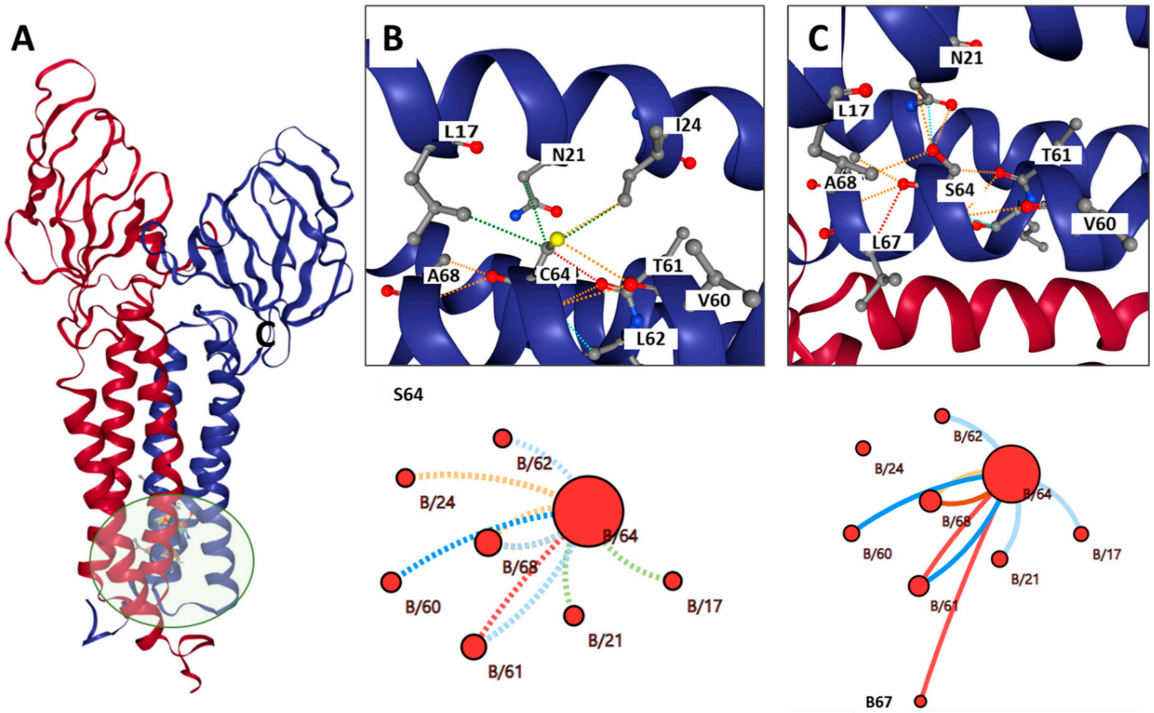

Figure S4. (A) Dimer model of membrane glycoprotein of SARS-CoV-2 (Blue and Brown). The model is adapted from (1). The circled region shows localization of C64S mutation. (A) Residue interaction of C64 for wild type membrane glycoprotein. Panel below shows detailed interaction of C64 with other residues of membrane glycoprotein (marked as Chain B, Dashed lines). (B) Residue interaction network for mutant S64 variant. Panel below shows detailed interaction of S64 with other residues of membrane glycoprotein (marked as Chain B, straight lines). Color codes: H-bonds (red), Polar H-bonds (orange), VdW (light blue), Aromatic (light green) and Ring-ring interactions (brown).

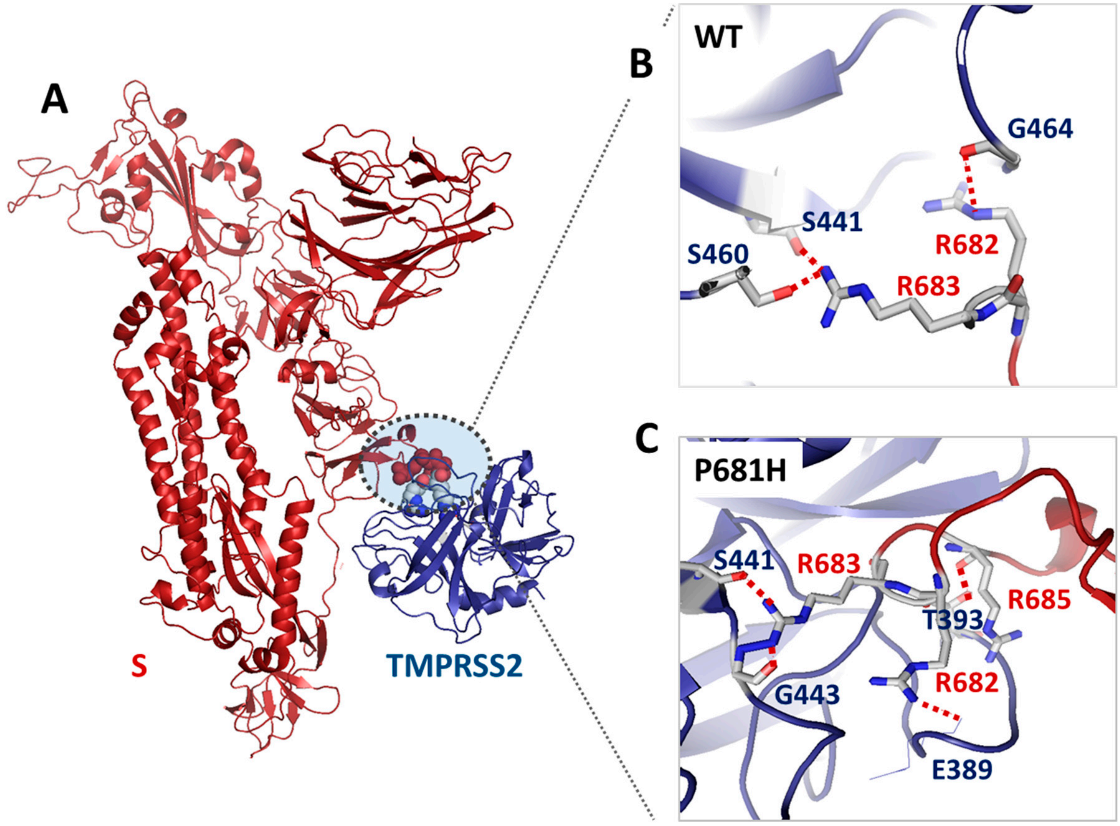

Figure S5. Structural analysis of P681H mutation near furin cleavage site. (A) Protein-protein docked model for S protein- TMPRSS2 complex with interaction interface at furin cleavage site. (B) Potential H-bond network for TMPRSS2-furin cleavage site (of wild type) interface. (C) Potential H-bond network for TMPRSS2-furin cleavage site (of P681H mutant) interface. The mutation has resulted in potentially higher H-bonds between TMPRSS and S protein at furin cleavage site.

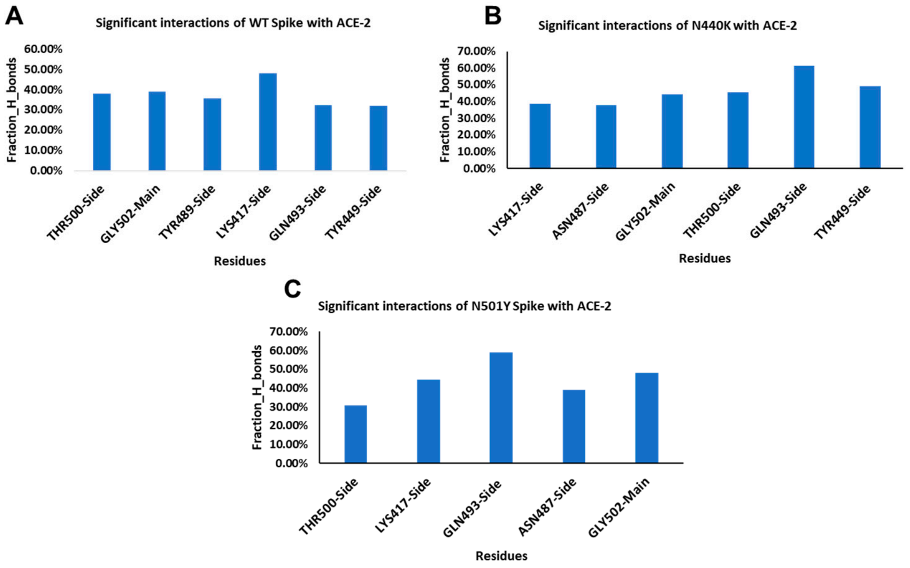

Figure S6. Interactive residues of Spike protein making significant hydrogen bond interactions with ACE-2. (A) Wildtype Spike-ACE2 (B) N440K Spike-ACE-2 (C) N501Y Spike-ACE2.

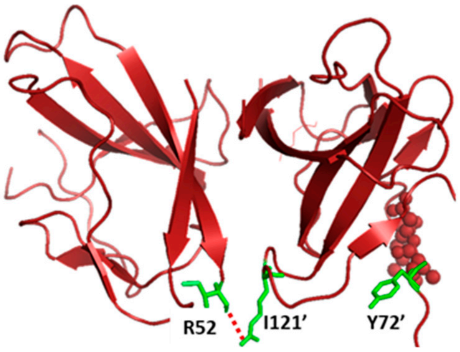

Figure S7. Cartoon representation of ORF8 (brown) dimer showing H-bond interactions of R52 and I121' (green sticks) between two monomers and Y72'.

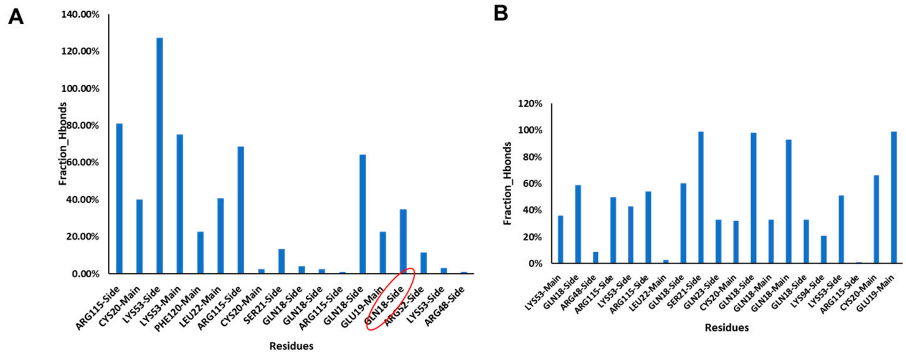

Figure S8. The hydrogen interaction fraction of the residues of ORF8 throughout the simulations. (A) ARG52 interactions in wildtype ORF8 was not significant as other residues. (B) In R52I mutant ORF, ILE52 was not making any significant hydrogen bond.

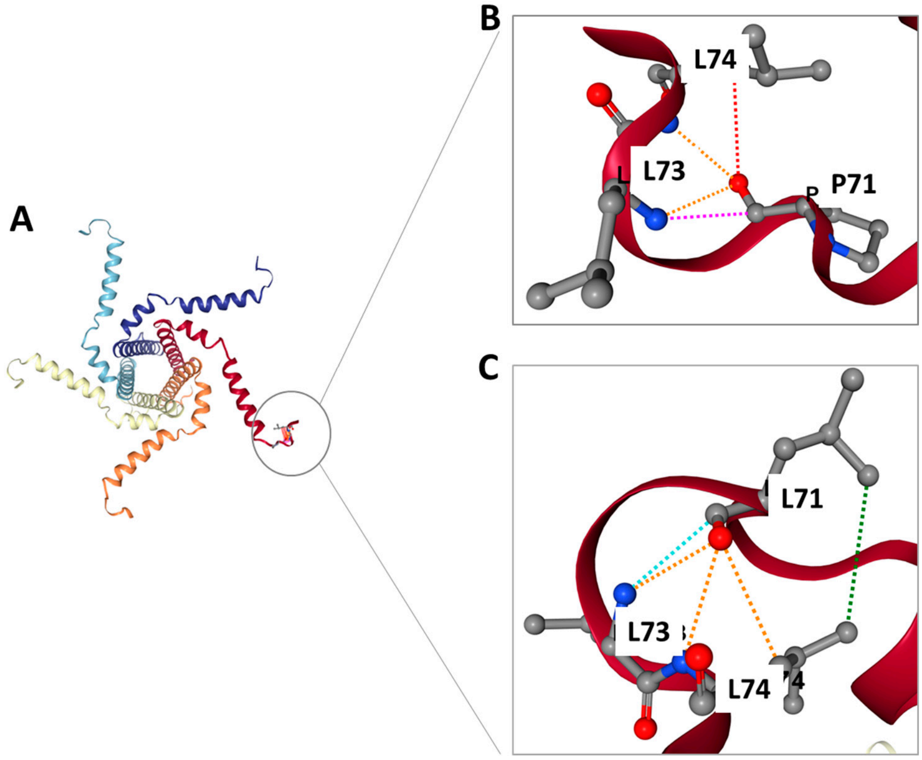

Figure S9. Structural analysis of P71L mutation in E protein. (A) Pentameric pore model of E protein. Adapted from [1] showing P71L mutation in its C-terminal domain (Circled) (B) Residue interaction network for wild type P71 variant. (C) Residue interaction network for mutant L71 variant. Color codes: H-bonds (red), Polar H-bonds (orange), VdW (light blue), Aromatic (light

green) and Ring-ring interactions (brown). The P71L mutation did not result in significant perturbation in local interaction network.

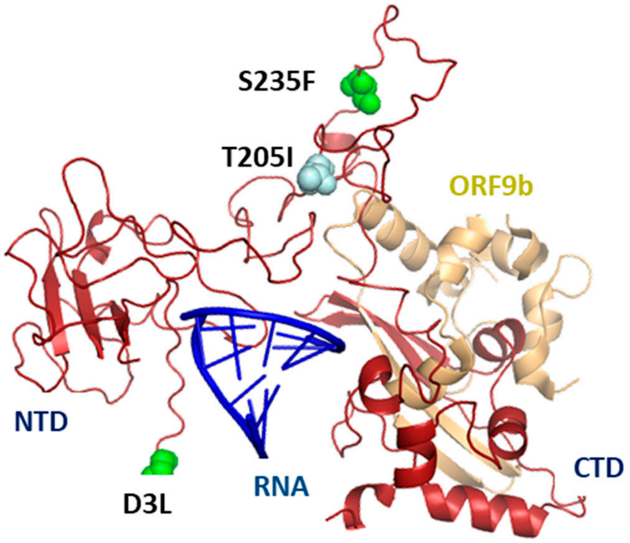

Figure S10. Cartoon representation of N protein RNA (blue) interacting NTD and ORF9b (golden yellow) interacting CTD. The two D3L, S235F (501Y.V1, Green spheres) and T205I (501Y.V2, cyan spheres) mutations are localized in the unstructured NTD and linker regions, respectively**.**

	Testing					Use
	Pre-clinical	*Phase I*	*Phase I/II*	*Phase II*	*Phase III*	*In use*
**RNA**	30	2	1	1	3	2
**DNA**	18	2	4		2	
**Non-replicating viral vector**	26	6			4	3
**Replication viral vector**	18	2	2	1		
**Inactivated**	10	1	1	1	6	4
**Live attenuated**	3	1				
**Protein subunit**	68	4	10	2	4	1
**Virus-like particle**	17		1		1	
**Other/Unknown**	33	2	3			

Figure S11. Summary of types of vaccines under various phases of clinical trials. As of January 9, 2021, 291 vaccine candidates are under clinical trials and 10 are in use.

Table S1. FireDock outcomes from protein-protein docking complex refinement of wildtype and mutant RBDs (S protein) with C135 and CR3022 antibodies. ACE and HB represents Atomic contact and H-bond interaction energies respectively.

Variant	Global energy		Attractive VdW		Repulsive VdW		ACE		HB	
	**C135**	**C3022**	**C135**	**C3022**	**C135**	**C3022**	**C135**	**C3022**	**C135**	**C3022**
**P1**	**4.01**	**-44.00**	-11.15	-24.66	0.66	10.88	4.77	-5.26	-2.60	-2.37
**B.1.1.7**	**1.43**	**- 43.32**	-12.26	- 23.08	1.94	5.39	4.28	-4.13	-3.45	-1.57
**B.1351**	**3.83**	**-44.56**	-11.93	-23.95	1.61	8.40	5.19	-4.83	-2.98	-3.51
**N440K**	**-2.17**	**-30.04**	-16.94	-20.26	6.24	4.15	3.17	-0.73	-4.21	-2.51
**S477N**	**3.90**	**-25.18**	-10.33	-20.47	0.98	7.62	4.36	0.09	-3.39	-3.33
**Wildtype**	**-17.03**	**-113.12**	-23.76	-61.07	7.15	14.93	7.32	-13.85	-3.67	-8.05

Table S2. DynaMut analysis for predicting impact of mutations of structural stability of proteins. Positive $\Delta\Delta G^{stability}$ SDM and $\Delta\Delta S_{Vib}$ ENCoM values indicate stabilizing mutations with increase in molecule flexibility. Negative values indicate destabilizing mutations with decrease in molecule flexibility. D215G, S982A and S235F were predicted to be highly stabilizing mutations in S and N proteins respectively. E484K and D3L were predicted to be mildly destabilizing mutations in S and N proteins respectively.

Gene	Mutation	ΔΔGstability SDM ($kcal.mol^{-1}$)	ΔΔSVib ENCoM ($kcal.mol^{-1}.K^{-1}$)
	D80A*	0.86	-0.2
S	ΔH69<br>ΔV70<br>ΔY144		
	D215G*	**1.91**	0.88
	K417N*	0.02	0.73
	K417T#	-0.95	-0.41
	E484K*#	**-0.41**	-0.28
	N501Y *#	**0.41**	-0.03
	A570D	0.21	0.10
	H655Y#	0.6	-1.35
	P681H	**0.76**	0.02

	A701V*		
	T716I	0.07	-0.34
	S982A	**1.56**	0.02
	T1027I#	**1.93**	-0.01
	D1118H	0.42	0.09
**S**	S477N (Australia)	0.31	-0.03
	N440K (India)	-0.13	0.04
**ORF8**	Q27Stop		
	R52I	0.82	1.244
	Y73C	-0.07	0.155
	E92K#	-0.58	0
**N**	D3L	-0.43	-0.67
	T205I*	0.14	0.51
	S235F	**1.867**	0.678
	P80R#	0.13	-0.19
**E**	P71L	0.04	-1.79

Table S3. Selected proposed drug candidates participating in key interactions with residues inside RBD of S protein of SARS-CoV-2.

Residue	Proposed Drugs	References
K417	Simeprevir, Lumacaftor, NPACT01552	(2, 3)
N440	Ledipasvir, Procyanidin, Strictinin, Saikosaponin E	(4)
S477	Evomonoside	(5)
E484	Paromomycin	(6)
N501	Grazoprevir, Acarbose	(4, 6)

Table S4. Various range of SARS-CoV-2 diagnostic tests with corresponding target genes.

S.No.	Detection Method	Target genes	Reference
1.	Antigen detection assay	Nucleocapsid (N gene) Spike protein (S gene)	(7)
2.	RT PCR test	E gene, N gene, S gene, RdRp gene and ORF1ab gene	(8)
3.	CRISPR based technique	N gene, Orf 1ab gene, S gene	(9)

	(Cas12a, Cas13, FnCas9)		
4.	RT-LAMP based assay	N gene, RdRp, S, ORF1a	(7, 10)
5.	COVID-19 CBNAAT based assay	E gene, RdRp gene, Orf-1a, N gene	(7, 11)
6.	Biosensor based kits	E, RdRp, ORF1ab, S	(10)
7.	ELISA-based antibody kits	N gene, S gene	(7)<br>(12)

References for Table 1 (Main manuscript): (13-19)

1. Portelli S, Olshansky M, Rodrigues CHM, D'Souza EN, Myung Y, Silk M, et al. Exploring the structural distribution of genetic variation in SARS-CoV-2 with the COVID-3D online resource. Nature genetics. 2020;52(10):999-1001.
2. Trezza A, Iovinelli D, Santucci A, Prischi F, Spiga O. An integrated drug repurposing strategy for the rapid identification of potential SARS-CoV-2 viral inhibitors. Scientific reports. 2020;10(1):13866.
3. Muhseen ZT, Hameed AR, Al-Hasani HMH, Tahir Ul Qamar M, Li G. Promising terpenes as SARS-CoV-2 spike receptor-binding domain (RBD) attachment inhibitors to the human ACE2 receptor: Integrated computational approach. Journal of molecular liquids. 2020;320:114493.
4. Kadioglu O, Saeed M, Greten H, Efferth T. Identification of novel compounds against three targets of SARS CoV-2 coronavirus by combined virtual screening and supervised machine learning: Bull World Health Organ; 2020 21 March 2020.
5. de Oliveira OV, Rocha GB, Paluch AS, Costa LT. Repurposing approved drugs as inhibitors of SARS-CoV-2 S-protein from molecular modeling and virtual screening. J Biomol Struct Dyn. 2020:1-10.

6. Tariq A, Mateen RM, Afzal MS, Saleem M. Paromomycin: A potential dual targeted drug effectively inhibits both spike (S1) and main protease of COVID-19. International journal of infectious diseases : IJID : official publication of the International Society for Infectious Diseases. 2020;98:166-75.
7. Kilic T, Weissleder R, Lee H. Molecular and Immunological Diagnostic Tests of COVID-19: Current Status and Challenges. iScience. 2020;23(8):101406.
8. Mahendiratta S, Batra G, Sarma P, Kumar H, Bansal S, Kumar S, et al. Molecular diagnosis of COVID-19 in different biologic matrix, their diagnostic validity and clinical relevance: A systematic review. Life sciences. 2020;258:118207.
9. Kumar P, Malik YS, Ganesh B, Rahangdale S, Saurabh S, Natesan S, et al. CRISPR-Cas System: An Approach With Potentials for COVID-19 Diagnosis and Therapeutics. Frontiers in cellular and infection microbiology. 2020;10:576875.
10. Ganguli A, Mostafa A, Berger J, Aydin MY, Sun F, Ramirez SAS, et al. Rapid isothermal amplification and portable detection system for SARS-CoV-2. Proceedings of the National Academy of Sciences of the United States of America. 2020;117(37):22727-35.
11. Zhen W, Smith E, Manji R, Schron D, Berry GJ. Clinical Evaluation of Three Sample-to-Answer Platforms for Detection of SARS-CoV-2. Journal of clinical microbiology. 2020;58(8).
12. Liu W, Liu L, Kou G, Zheng Y, Ding Y, Ni W, et al. Evaluation of Nucleocapsid and Spike Protein-Based Enzyme-Linked Immunosorbent Assays for Detecting Antibodies against SARS-CoV-2. Journal of clinical microbiology. 2020;58(6).
13. Kemp SA, Datir RP, Collier DA, Ferreira I, Carabelli A, Harvey W, et al. Recurrent emergence and transmission of a SARS-CoV-2 Spike deletion ΔH69/ΔV70. bioRxiv : the preprint server for biology. 2020:2020.12.14.422555.
14. McCarthy KR, Rennick LJ, Nambulli S, Robinson-McCarthy LR, Bain WG, Haidar G, et al. Natural deletions in the SARS-CoV-2 spike glycoprotein drive antibody escape. bioRxiv : the preprint server for biology. 2020:2020.11.19.389916.
15. Fratev F. The N501Y and K417N mutations in the spike protein of SARS-CoV-2 alter the interactions with both hACE2 and human derived antibody: A Free energy of perturbation study. bioRxiv : the preprint server for biology. 2020:2020.12.23.424283.
16. Chen J, Wang R, Wang M, Wei G-W. Mutations Strengthened SARS-CoV-2 Infectivity. J Mol Biol. 2020;432(19):5212-26.
17. Chen J, Gao K, Wang R, Wei G. Prediction and mitigation of mutation threats to COVID-19 vaccines and antibody therapies. ArXiv. 2020.
18. Weisblum Y, Schmidt F, Zhang F, DaSilva J, Poston D, Lorenzi JCC, et al. Escape from neutralizing antibodies by SARS-CoV-2 spike protein variants. bioRxiv : the preprint server for biology. 2020:2020.07.21.214759.
19. Starr TN, Greaney AJ, Hilton SK, Ellis D, Crawford KHD, Dingens AS, et al. Deep Mutational Scanning of SARS-CoV-2 Receptor Binding Domain Reveals Constraints on Folding and ACE2 Binding. Cell. 2020;182(5):1295-310 e20.
